# Perceived stress of COVID-19 pandemic and problematic mobile phone use during quarantine conditions among Chinese adolescents: a mediated moderation model

**DOI:** 10.3389/fpsyg.2024.1333869

**Published:** 2024-02-01

**Authors:** Shuyang Jiang, Lifan Zhang

**Affiliations:** ^1^Department of Psychology, Suzhou University of Science and Technology, Suzhou, China; ^2^School of Education, Yunnan Minzu University, Yunnan, China

**Keywords:** perceived stress, problematic mobile phone use, search for meaning in life, escapism motivation, adolescents

## Abstract

The relation between perceived general stress and problematic mobile phone use (PMPU) has been well established. With the outbreak of the coronavirus disease (COVID-19), the present study was designed to examine the association between perceived stress of COVID-19 as a kind of event-related stress and PMPU, and the mechanisms underlying this relation. Participants were 724 adolescents ranging from 12 to 16 years old (*M* = 13.28, *SD* = 1.05) who completed four online questionnaires addressing perceived stress of COVID-19, search for meaning in life, escapism motivation, and PMPU. The results revealed that escapism motivation mediated the relationship between perceived stress of COVID-19 and PMPU. In addition, search for meaning in life played a moderating role between perceived stress of COVID-19 and escapism motivation. These findings extend the literature by addressing how and under what conditions perceived stress of COVID-19 can contribute to PMPU. We discussed the implications for developing targeted intervention programs aimed at reducing PMPU among adolescents.

## 1 Introduction

With the rapid development of mobile internet, mobile phones have become an integral part of people’s lives, especially among adolescents in China (Yang et al., 2019; Zhang et al., 2022). As digital natives, while there are psychosocial benefits linked to mobile phone use (Schneider et al., 2023), excessive usage may result in problematic mobile phone use (PMPU), which is characterized by a series of mobile phone addiction symptoms, including withdrawal, craving, functional impairment, and associated deleterious consequences (Chang and Ko, 2023; Ng et al., 2020). While the Diagnostic and Statistical Manual of Mental Disorders, Fifth Edition (DSM-5) does not include a mobile phone-specific addiction disorder (American Psychiatric Association, 2013). PMPU can result in negative consequences in academic performance (Kates et al., 2018); physical and mental health, including bodily pain, sleep disturbance, symptoms of depression, anxiety (Ng et al., 2020); as well as social and interpersonal difficulties (Tang et al., 2018).

According to the General Strain Theory (Agnew, 1992), negative experiences related to stress serve as the primary determinants of problematic behaviors. With the outbreak of the COVID-19 pandemic, individuals encountered significant threats to their health and safety (Kozina et al., 2021). In China, schools were suspended, leading adolescents to adapt to a new routine characterized by remote learning from home. As they transitioned from a structured school environment to a relatively flexible, self-determined lifestyle, mobile phones became increasingly vital for information retrieval, communication, and entertainment, serving as a portable “first aid” to cope with stressful situations (Schneider et al., 2023). According to the model of compensatory internet use (Kardefelt-Winther, 2014a,b), individuals turn to online activities to cope with offline stressors, suggesting that the desire to seek escape from reality is a key driver of usage behavior. With the goal of integrating escapism motivation into the stress-PMPU connection, we examine whether the escapism motivation play a possible mediator in the connection between perceived stress of COVID-19 and PMPU.

In the context of significant safety crises and lifestyle changes, individual characteristics associated with life-meaning system may interact with stress coping (Borawski et al., 2021). Search for meaning acts as a scheme that directs attention towards meaning-related information (Steger et al., 2011), implying that search for meaning may strengthen the negative effects of stressors on coping process. Especially in situations like life-threatening public emergency (i.e., COVID-19 pandemic), it becomes crucial to examine whether the searching for meaning influences how individuals interpret and adapt to the challenges presented by the circumstances.

This study aimed to fill these gaps by investigating whether perceived stress of COVID-19, as an event-related stress, contributes to PMPU among Chinese adolescents during the pandemic-related quarantine. Additionally, we examined the mediating role of escapism motivation and the moderating effect of searching for meaning in life.

### 1.1 The association between perceived stress of COVID-19 and PMPU

Previous research has reported life stress as one of the major causes of problematic behaviors such as PMPU and substance addiction (Patterson et al., 2021). Stress can be defined as a response state that occurs when individuals perceive that a situation is threatening or challenging and that they lack the resources to combat the stressor (Wang et al., 2015). According to the general strain theory (Agnew, 1992), high stress is often associated with various psychological symptoms, and mobile phones may serve as “first aid in the pocket” to cope with daily stressors (Schneider et al., 2023), thereby increasing the risk of PMPU (Ying et al., 2022).

In addition to daily life stress, event-specific stress, such as the perceived stress of COVID-19, may correlate with PMPU. Life-threatening events, such as illnesses and disasters, are among the most powerful contextual stressors that can elevate feelings of nervousness and perceived stress, consequently leading to behavioral changes (Leung, 2007; Qiu et al., 2020). Yang et al. (2020) conducted a study with a sample of 776 adolescents who experienced a major earthquake, and the results showed that post-traumatic stress symptoms had positive predictive effects on internet addiction. In relation to the COVID-19 pandemic, recent studies have indicated that COVID-19 can trigger excessive stress and negative emotions, leading to an increase in substance use (Patterson Silver Wolf, 2020; Patterson et al., 2021). Considering the established association between general stress, PMPU, and the stressful nature of the coronavirus disease, it seems logical to assume that the perceived stress of COVID-19 might contribute to adolescents’ PMPU.

### 1.2 Escapism motivation as a potential mediator

According to the model of compensatory internet use (Kardefelt-Winther, 2014a,b), people spend time online to cope with real-life issues, and the motivations for such behavior are grounded in psychosocial problems. From this perspective, escapism motivation, which refers to one’s need to escape from reality and use their mobile phone as a means to do so due to encountering setbacks in real life (Kim, 2017), may potentially mediate the pathway from perceived stress of COVID-19 to PMPU.

Social Cognitive Theory (Bandura, 1989) provides theoretical guidelines for explaining the mediating role of escapism motivation. Suffering from stress involves both negative emotions (e.g., anxiety, depression) and low controllability (Qiu et al., 2020), which are closely connected to negative coping strategies (Kozina et al., 2021; Patterson et al., 2021), leading individuals to be more inclined to seek escapism as a way to alleviate stress. Recent studies showed that perceived stress of COVID-19 can induce negative affect and decrease the resources that are necessary for self-regulation (Qiu et al., 2020; Kozina et al., 2021).

As a refuge from reality, mobile phone usage frequently emerges as a favorable avenue for managing psychological distress and intense stress (Leung, 2007; Kardefelt-Winther, 2014a; Wang et al., 2015). Nevertheless, while mobile phone use offers a momentary escape from reality, such instant gratification may contribute to the onset of mobile phone addiction. Studies have found that people who use mobile phones to escape from reality are often at higher risk of developing PMPU (Caplan et al., 2009; Wang et al., 2015). Taken together, these findings support the notion that adolescents with higher levels of perceived stress associated with COVID-19 to be more inclined to use mobile phones to escape from their current negative mental state and to be more likely to engage in PMPU.

### 1.3 Search for meaning in life as a potential moderator

Research evidence has consistently underscored the interaction between individuals and their environment, highlighting individual differences in susceptibility to environmental influences (Zhuang et al., 2017; Ying et al., 2022). Facing significant public safety threats, people may react differently depending on their goal-driven value system, such as meaning in life (Steger et al., 2008; Louws-Hendriksen et al., 2022). Meaning in life is consisted by two related yet distinct constructs, including presence of meaning and search for meaning (Wilt et al., 2017). The function of presence of meaning has yielded consistent results, affirming its buffering effect against stress (Copeland et al., 2020). However, the role of search for meaning appears to be less clear (Borawski et al., 2021).

Searching for meaning in life reflects individuals’ level of effort in the pursuit of establishing and enriching their comprehension of the meaning, significance, and purpose of their lives (Steger et al., 2008). On the one hand, the majority of previous studies has shown that meaning-seeking to be closely associated with negative views of self, negative emotions (e.g., anxiety, depression) and passive thinking (Wilt et al., 2017; Copeland et al., 2020), all of which increase the vulnerability in stressful situations (Louws-Hendriksen et al., 2022). On the other hand, some studies indicated that searchers exhibit greater curiosity and openness to their experiences, thereby may have potential benefits (Yang et al., 2019). These inconsistent findings may be explained by the differences in the valence of the events involved. Search for meaning acts as a scheme that organizes and directs attention towards information which is relevant to meaning in life (Steger et al., 2011). That is, it strengthens any meaning-related experience, regardless of its valence (Borawski et al., 2021).

Following this logic, it is possible to assume that search for meaning in life may exert an amplifier effect in the link between perceived stress of COVID-19 and escapism motivation. Considering the context of COVID-19，adolescents who scored higher on search for meaning are more likely to engage in rumination regarding the disappointing news and unpleasant experiences during home quarantine (Steger et al., 2008, 2011), and thus they may be more likely to turn to mobile phones as a temporary escape (Leung, 2007; Wang et al., 2015). Based on the above evidence, we speculated that search for meaning would moderate the relation between perceived stress of COVID-19 and escapism motivation, with this effect being stronger for individuals with higher levels of search for meaning in life.

### 1.4 The current study

**Figure 1:**
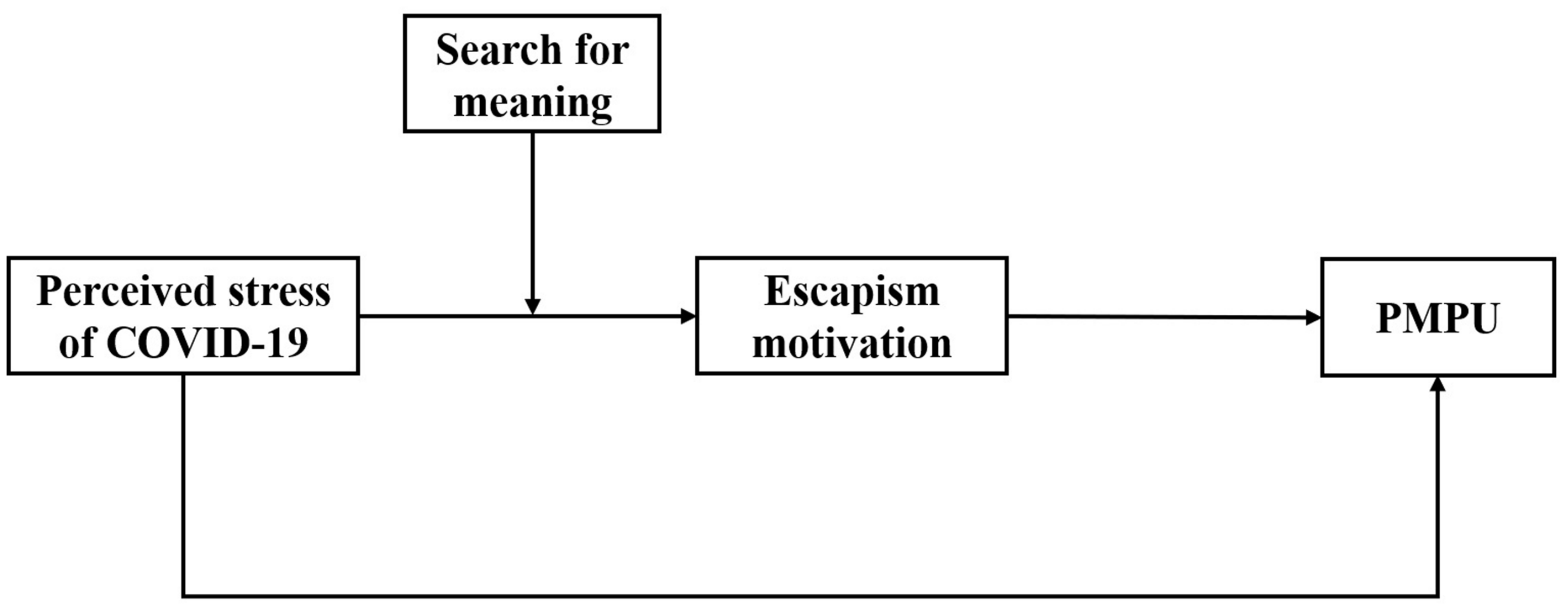
Conceptual model. *Note*. PMPU = problematic mobile phone use.

In the present study, we constructed a moderated mediation model to shed light on why (mediation) and under what conditions (moderation) perceived stress of the COVID-19 was related to PMPU among Chinese adolescents during quarantine conditions (see Figure 1). The following hypotheses were addressed.

H1: Perceived stress of COVID-19 positively relates to PMPU.

H2: Escapism motivation mediates the path from perceived stress of COVID-19 to PMPU. Specifically, perceived stress of COVID-19 positively predicts escapism motivation, and escapism motivation positively predicts PMPU.

H3: Search for meaning in life acts as a moderator in the relation between perceived stress of COVID-19 and escapism motivation. Specifically, the connection between perceived stress of COVID-19 and escapism motivation is amplified when the degree of search for meaning in life increases.

The findings contribute to the existing literature by (a) providing a more thorough understanding of why students with a heightened perception of event-related stress exhibit higher levels of PMPU, and (b) validate the moderating role of meaning-seeking to demonstrate the interaction between individuals’ life meaning system and stress coping process. These insights provide valuable guidance for intervention practices targeted at individuals undergoing stress events.

## 2 Method

### 2.1 Participants

Recruited from two public middle schools in Guangdong, China, a total of 781 students participated in the study. From them, we obtained 724 (M = 13.28, SD =1.05, 49.9% males) valid questionnaires, the response rate was 92.7%. All participants identified themselves as mobile phone users.

### 2.2 Measures

#### 2.2.1 Perceived stress of COVID-19 epidemic

A five-item scale was used to capture participants’ degree of perceived stress associated with the COVID-19 epidemic. The scale was adopted from Antony et al. (1998), and was found to be reliable among Chinese sample (Cao et al., 2023). To capture the specific context of the pandemic, we modified it by adding “Because of the COVID-19’’ at the beginning of relevant items (see Jiang et al., 2020 for a similar approach). A sample item is, “Because of COVID-19, I find it difficult to relax.” Students rated the items on a 5-point Likert scale ranging from 1 (*not true at all*) to 5 (*very true*). The scale showed good reliability in the current study (Cronbach’s alpha reliability coefficient = 0.946).

#### 2.2.2 Search for meaning

Participants’ search for meaning was assessed using the search for meaning subscale from the Meaning in Life Questionnaire (MLQ-S; Steger et al., 2006). A sample item is, “I am seeking a purpose or mission for my life.” Students were asked to choose the extent to which each item applied to them using a 7-point scale ranging from 1 (*absolutely untrue*) to 7 (*absolutely true*). The Chinese version of the MIQ-S had demonstrated good reliability and construct validity (Wang, 2013), as well in this study α = 0.873.

#### 2.2.3 Escapism motivation

The degree of escapism motivation related to mobile phone use was assessed using a Chinese translated version of the escapism motivation scale developed by Kim (2017). Participants were asked to indicate how strongly they agreed with the listed motivations for using a mobile phone. A sample item is, “I use/play with my mobile phone to forget about heavy school assignments.” Students were asked to respond on a 5-point Likert scale ranging from 1 (*strongly disagree*) to 5 (*strongly agree*). The items were found to be reliable and valid in prior research among Chinese populations (Chai et al., 2022). The internal reliability in the present study was 0.867.

#### 2.2.4 Problematic mobile phone use

The degree of students’ PMPU was rated with a 10-item mobile phone problem use scale (Foerster et al., 2015). A Chinese version that proved to be valid among Chinese adolescents was adopted to fit the context of the COVID-19 outbreak (Zhuang et al., 2017). A sample item is, “During the peak time of COVID-19, I find it difficult to switch off my mobile phone.” Participants responded on a 5-point scale ranging from 1 (*strongly disagree*) to 5 (*strongly agree*), with higher scores indicating a higher degree of PMPU. The present sample revealed a good internal consistency (Cronbach’s *α* = 0. 872).

### 2.3 Procedure

Ethical approval for carrying out this study was obtained from a major research university in China. We contacted school administrators to explain our research purpose and asked for their permission to conduct the study. Teachers, students, and their parents were fully informed about the study objectives and the confidential nature of the study. Students’ assent and digital consents from their parents were obtained. Participants completed a self-report questionnaire using an online survey system that took approximately 15 min.

### 2.4 Data analysis plan

Before testing our hypotheses, a one-factor test was conducted to check the common method variance using SPSS 23.0 software. Descriptive statistics and Pearson correlations coefficients between the main variables were calculated.

To test the hypotheses, we used the bootstrapping method with PROCESS macro^1^ to calculate the 95% confidence intervals with 5,000 resamples (Hayes, 2013).

## 3 Results

### 3.1 Common method variance analysis

Before testing the hypotheses, we assessed the common method variance (CMV) by conducting Harman’s one-factor test. If one general factor accounts for more than 40% of the total variances, it indicates the presence of a common method variance. In the present study, the EFA results extracted seven factors with eigenvalues exceeding 1, and the first factor explained 29.26% of the total variance, which indicated that common method variance was not a serious concern in the present study.

### 3.2 Means, standard deviations, and correlations among variables

Table 1 displays the descriptive statistics and bivariate correlations among the variables. The degree of search for meaning was significantly and positively correlated with perceived stress of COVID-19, escapism motivation, and PMPU. In addition, correlations among perceived stress of COVID-19, escapism motivation, and PMPU were significant and positive. Furthermore, age was found to positively correlate with PMPU and was controlled as a covariate in further analyses.

**Table 1:** Means, standard deviations, and correlations among variables.

	*M* ± *SD*	1	2	3	4	5	6
1. Age	13.288 ± 1.051	-					
2. Gender	-	-	-				
3. Search for meaning	5.383 ± 1.365	0.139^**^	0.036	-			
4. Perceived Stress of COVID-19	3.073 ± 1.865	0.007	−0.055	0.203^***^	-		
5. Escapism motivation	3.090 ± 1.186	−0.007	0.011	0.090^*^	0.376^***^	-	
6. PMPU	2.672 ± 0.955	0.147^**^	−0.04	0.107^*^	0.494^***^	0.641^***^	-

Gender coded as 1 = male; 2 = female; PMPU = problematic mobile phone use.

∗ *p* < 0.05, ∗∗ *p* < 0.01, ∗∗∗ *p* < 0.001.

### 3.3 Testing the mediation of escapism motivation

To test the mediation model, after controlling for age, we placed escapism motivation as a mediating variable in the relation between perceived stress of COVID-19 and PMPU. The process macro (M4) was used to examine this model.

The results revealed that perceived stress of COVID-19 had a positive significant effect on escapism motivation (path a; *β* = 0.239, *p* < 0.001, 95% CI [0.186, 0.291]) and on PMPU (path c′; *β* = 0.150, *p* < 0.001, 95% CI [0.115, 0.185]), while escapism motivation had a positive effect on PMPU (path b; *β* = 0. 428, 95% CI [0.373, 0.484]), supporting both H1 and H2.

### 3.4 Moderated mediation analyses

**Figure 2:**
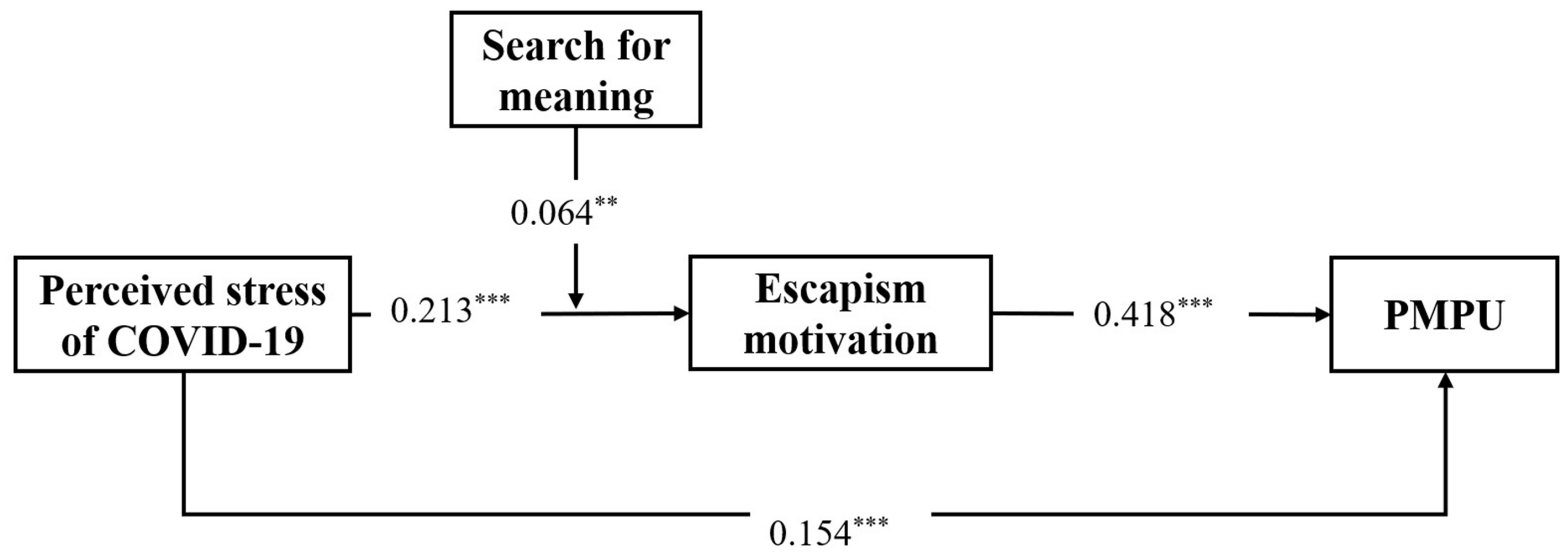
The moderated mediation model after controlling for age. *Note*. PMPU = problematic mobile phone use. ∗∗ *p* < 0.01, ∗∗ *p* < 0.001.

To test the moderating effect of search for meaning in the hypothesized model, PROCESS macro (M59) was used; the results are displayed in Figure 2. The results revealed that the interaction term could significantly predict escapism motivation (*β* = 0. 064). Additionally, the results of the 95% confidence interval from 5,000 bootstrap samples showed a range from 0.022 to 0.1070, which does not include zero, indicating that search for meaning in life could moderate the relation between perceived stress of COVID-19 and escapism motivation.

**Figure 3:**
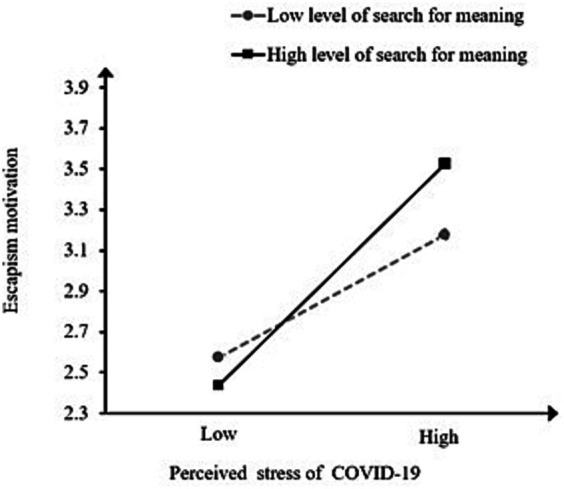
The interaction of search for meaning and perceived stress of COVID-19 on escapism motivation.

To further reveal the pattern of the interaction, a simple slope analysis was carried out. Conditional effects of perceived stress of COVID-19 on escapism motivation under different levels of search for meaning (M ± SD) were plotted respectively; see Figure 3. The findings revealed that with a high level of search for meaning, perceived stress of COVID-19 could predict escapism motivation (*β* = 0. 301, *p* < 0.000); with a low level of search for meaning, the prediction of perceived stress of COVID-19 was also significant (*β* = 0. 125, *p* < 0.01). These results indicated that the conditional effects of perceived stress of COVID-19 on escapism motivation were significantly larger for students with a high level of search for meaning, which supported H3.

## 4 Discussion

Our study aimed to examine whether escapism motivation mediated the relationship between perceived stress of COVID-19 and PMPU, and whether search for meaning in life was a moderator between perceived stress of COVID-19 and mediator. Not only did we integrated the general strain theory and the model of compensatory internet use to demonstrate that PMPU is a result of negative stress coping, we also tried to clarify under what conditions the perceived stress varied with different individual characteristics. Our results indicated that escapism motivation served a mediating role in the perceived stress of COVID-19-PMPU link, and search for meaning moderated the association between perceived stress of COVID-19 and escapism motivation.

### 4.1 Perceived stress of COVID-19 and PMPU

In line with H1, the results of the direct effect revealed a strong relationship between perceived stress of COVID-19 and PMPU. This finding concurs with the general strain theory (Agnew, 1992) and previous research (Liu et al., 2018; Chang and Ko, 2023), suggesting that problematic behaviors may occur as a result of stress caused by real-life situation. Moreover, our finding consists with other studies conducted in the context of the COVID-19 pandemic (Patterson et al., 2021; Yam et al., 2021). For example, Fernandes et al. (2020) examined the impact of lockdown on internet use among adolescents from several countries (e.g., India, Malaysia, Mexico, UK), comparing their habits from before the pandemic, and showed that adolescents have generally reported high scores of negative emotions (e.g., depression, anxiety) and increased use of internet services.

As a worldwide public health emergency, the COVID-19 outbreak has affected not only people’s physical health but also their daily lives, as governments adopt active measures (e.g., travel restrictions, social distancing, remote learning) to prevent the spread of the virus. Such drastic societal changes can induce significant stress, especially among adolescents, who are mostly forced to stay home and transition to remote learning. Adolescents who scored high on perceived stress are prone to use mobile phones to pass the time, eliminate negative emotions, and relieve stress (Patterson et al., 2021), thus making them more vulnerable to PMPU (Yam et al., 2021). Combining the results of this study with the aforementioned research, the findings support the view that, regardless of the country of residence, the COVID-19 outbreak had a significant impact on adolescents’ maladaptive coping strategies, such as PMPU.

### 4.2 The mediating role of escapism motivation

Our findings regarding the mediating role of escapism motivation supported H2, that is, escapism motivation served as a mediator for interpreting how perceived stress of COVID-19 was linked to increased PMPU. These findings support the compensatory internet use theory (Kardefelt-Winther, 2014a), when adolescents perceived high levels of stress associated with COVID-19, they are more likely to rely on mobile phone services, such as internet gaming, to distract their attention away from negative experiences associated with the pandemic. Furthermore, studies have demonstrated that individuals who rely on mobile phone use to escape from real-life problems or to alleviate negative feelings not only are unable to relieve their original issues through media but also are more vulnerable to PMPU (Balhara et al., 2020).

These results call for special attention. Suffering from the distress of the pandemic, adolescents often struggle to adopt proactive coping strategies, leading to a predominant desire for escapism as a coping mechanism. In alignment with the “poor-get-poorer” perspective (Stevic and Matthes, 2021), our results suggest that individuals with existing psychological challenges are less likely to benefit from media experiences, as they may exchange one problem (perceived stress) for another (i.e., PMPU).

### 4.3 The moderating role of searching for meaning in life

Consistent with H3 and prior research demonstrating the maladaptive functions associated with search for meaning (Bodner et al., 2013; Wilt et al., 2017), we found that search for meaning played a moderating role in the association between perceived stress of COVID-19 and escapism motivation. For adolescents who actively seek for meaning in life, they may view the experience of the pandemic as unpredictable, uncontrollable and overloaded (Steger et al., 2011). Such adverse experience not only increase the vulnerability of adolescents, but also interfere with self-regulation process (Bodner et al., 2013; Wilt et al., 2017), resulting in increased escapism motivation. Our result coincides with the risk-magnifying effect, which implies that search for meaning may serve as a stress-amplifier when facing severe life events.

Furthermore, our finding lends support to the “dual nature” underlying the search for meaning (Steger et al., 2008; Borawski et al., 2021). In addition to the evidence highlighting the negative effects associated with meaning-seeking, it is important to note that some studies have documented its potential to attenuate the impact of negative experiences. For instance, Yang et al. (2019) found that search for meaning could buffer the association between boredom proneness and depression. As mentioned previously, differences in valence (such as controllability and severity) of the contents, may account for these inconsistent results. Compared to the COVID-19, boredom proneness is relatively momentary and controllable that individuals can regulate their activities instead of being immersed in the adverse experience (Yang et al., 2019). To conclude, search for meaning can be considered a susceptibility factor, and its impact depends on specific situational characteristics.

## 5 Conclusion

The current study investigated the underlying mechanisms accounting for the relationships between perceived stress of the COVID-19 and PMPU among Chinese adolescents. Adolescents’ stress perception could positively predict PMPU not only through a direct path, but also through an indirect path via escapism motivation. Searching for meaning in life exerts an amplifier effect in the link between perceived stress of COVID-19 and escapism motivation. The present study enriches our understanding of why and how adolescents with a heightened perception of event-related stress exhibit higher levels of PMPU. Meanwhile, the findings expand theories related to the meaning in life by demonstrating that meaning-seeking enhances individuals’ vulnerability when facing significant public events. These findings offer valuable insights for developing interventions aimed at individuals experiencing stressful events.

## 6 Limitations and implications

Several limitations of the present study should be noted. First, the results of the present study indicated that perceived stress of COVID-19 was positively associated with PMPU. However, given that this study was based on cross-sectional data, further research is needed to determine whether this relation remains robust as the circumstances change. Second, all measures were self-reported by participants, which limits the generalizability of our findings. Future research could consider collecting data using multiple sources and methods of evaluation (e.g., behavioral criteria), to extend our findings. Moreover, the present study mainly focused on the moderating role of search for meaning in the stress coping process. Future researchers could examine whether the experience of the pandemic would influence individual’s life meaning system.

In spite of these limitations, the current study illuminates how individuals’ perception of stress contributes to their coping strategies and behavioral responses. These results have important applications with regard to the development of targeted interventions to reduce students’ PMPU. First, considering the negative impact of perceived stress of COVID-19 on PMPU, more attention should be given to individuals who are undergoing significant stressful events, such as those at high risk of infection. Meanwhile, the mediating role of escapism motivation implies that programs aimed at developing stress coping strategies can be useful in protecting students from PMPU. Targeted interventions can be instrumental in reducing maladaptive patterns of mobile phone use (Lin et al., 2018). For example, participating in physical activity can be helpful in reducing adolescents’ mobile phone dependence by improving self-control (Zhang et al., 2022). Finally, our results suggest that when confronted with significant real-life stressors, adolescents who actively search for meaning are at higher risk of maladjustment. Psychoeducational programs focusing on enhancing coping strategies, fostering resilience, and promoting a better understanding of the relationship between stressful events and life meaning might be helpful in harnessing the positive aspects of meaning-seeking (van Doornik et al., 2023).

## Data availability statement

The raw data supporting the conclusions of this article will be made available by the authors, without undue reservation.

## Ethics statement

The studies involving humans were approved by Ethics Committee of Beijing Normal University (IRB Number: 202106040033) and the principals of the participating schools. The studies were conducted in accordance with the local legislation and institutional requirements. Written informed consent for participation in this study was provided by the participants' legal guardians/next of kin.

## Author contributions

All the coauthors are participants in the data collection, data analysis, writing and revising the manuscript.

## Footnotes

^1^ http://www.afhayes.com
